# A transitivity analysis of Hillary Clinton and Donald Trump’s third presidential debate

**DOI:** 10.1016/j.heliyon.2022.e10518

**Published:** 2022-09-09

**Authors:** Farah Kashif, Rabia Farooqi, Shahnila Tariq, Aasia Nusrat, Farzana Ashraf, Abdullah Raees

**Affiliations:** a Kinnaird College for Women University, Lahore, Pakistan; b Department of Psychology, University of Central Punjab, Lahore, Pakistan; c Department of Applied Psychology UMT, Lahore, Pakistan; d COMSATS University Islamabad, Lahore Campus Lahore, Pakistan; e University of Lahore, Lahore, Pakistan

**Keywords:** American Presidential debates, Systemic functional grammar, Underlying Stimuli and Smart Strategies, Transitivity choices

## Abstract

This paper investigates the language of the last of the three American presidential debates between Hillary Clinton and Donald Trump before the 2016 general election. To explore and interpret the process-choices within verbal groups from the perspective of systemic functional grammar, it used a transitivity toolkit. This paper aims to identify the underlying stimuli and smart strategies from the frequency of transitivity choices. The meaning of these choices was investigated through ideational meta-functions which reflect the experiences of text producers using a mixed method approach. The comparative textual analysis of the sample text found that Donald Trump used fifty-one percent whereas Hillary Clinton used forty-nine percent of the processes in the debate. The predominant use of mental, behavioral, relational and existential process types by Hillary Clinton revealed her cognitive, sentimental, sensitive, attributive and existential approach. The frequent use of material and verbal process types by Donald Trump reflected his explicit and tangible outlook on the key issues facing the American state. The results also illustrate that the varying frequency of transitivity choices by both political rivals chiefly aimed at winning the support of the public in the polls.

## 1 Introduction

Halliday (1994) argues that an interesting feature of language use is that it helps text producers invoke an idiosyncratic image of a phenomenon. These semantically infused images are communicated through the structure of segments which can be decoded to reveal meaning beyond the sentence level. Hence, the meaning content of the third American presidential debate has been explored through its transitivity system to identify the implicit reasons and the explicit functions that stimulate their use.

Of the many genres of discourse, political discourse provides a specially rich ground for linguistic analysis because of the fact that politicians’ power-hunting tactics, generally, manipulate language for a careful representation, and at the same time distortion of, the ideology they aim at expressing in their speeches and public statements. The road that leads to the hearts and minds of public is language. Multiple functions are performed through linguistic choices. As Campbell (2008) asserts that in political speeches and presidential debates, communicative targets are varied enough to achieve diverse range of outcomes and functions. Well-calculated moves in the form of transitivity choices by American presidential rivals overwhelm the thinking capacities of public and mould them according to their will for reaching the echelons of power. Experiential value of their choices in internal as well as external world is paramount for in this way readers come to know about past incidents of political leaders of their country.

American presidential debates in general and the one between Hillary Clinton and Donald Trump in particular, just ahead of 2016 general elections, attracts researcher's attention for its text's abounding intricacies related to process types as laid down by Halliday in his Systemic functional Grammar. Halliday's focus has always been on the implications of language in use in the textual processes of social life or the socio-semantics of text.

This article, within the genre of political discourse, examines the transitivity choices made by Hillary Clinton and Donald Trump in the third presidential debate of the 2016 general election in America.

Taking a Systemic Functional Grammar approach, the present study explores the variations in the frequency of process types to uncover the reasons for their use an their functions in communicating the intentions of the speakers. The present study aids masses of the world in general and of America in particular in providing an orientation of subsequent political decades through textual analysis of debate between Hillary Clinton and Donald Trump

The variation of frequency in the process-type choices made by Hillary Clinton and Donald Trump during the debate reflect their outlooks on the six key issues discussed in the debate as well as serving the presidential candidates' political ends. The six issues are: the Supreme Court; immigration; the economy; candidates’ fitness for the Presidency; foreign hotspots; the national debt. The present study has compared and analyzed the transitivity choices made by each candidate in each area of debate identified above and explored the reasons for their choices as well as the impact they had on voters.

According to Halliday (1985), a system-network is a theory of language as choice. ‘Systemic’ in the SFL refers to the system of choices. Extensive reliance of speakers on particular choices guides the researcher to draw well-reasoned inferences and conclusions. Choices within communicative events like the last American presidential debate of 2016 were made by the speakers considering their needs, priorities and aims. Systemic Functional Grammar regards the relation of transitivity not with verbs alone, but with the whole clause in which they appear. While verbs always play a key role in creating meaning and making any group of words meaningful, in the SFL model, the system of transitivity is concerned with the participants, processes and circumstances as well.

While an exploration of the transitivity choices in the American presidential discourse has been attempted by researchers (e.g. Zhang, 2017; Mengyan, 2017; Junling, 2010; Mengyn Zhao & Yi Zhang, 2017; Zhu Yujie & Li Fengjie, 2018) no comparative study of the variation in therequency of process-type choices (material, mental, relational, verbal, existential or behavioral), the likely reasons for them and their functions in the third and final presidential debate between Hillary Clinton and Donald Trump has been undertaken so far.

The current research aims to focus on only one key concept of transitivity from the SFL model to know about the reasons that stimulated Hillary and Trump's choices and their resulting effects. This key concept of transitivity is one of several levels of textual analysis which provides the answers to two pertinent questions as to why does a communicative event takes place and what it is about. The reasons of fluctuation in the use of processes thus become evident. The major reason of choosing only the concept of transitivity in the systemic functional grammar framework is because the relationships of Donald Trump and Hillary Clinton with their countrymen and their respective perspectives on pertinent pre-poll issues can be studied by analyzing the inter-relation of processes with roles and circumstances. As such, it helps achieve the goals of the study.

The study focused the following research questions:

1. How frequently do the six different process types occur in each area of the debate?
2. Why do certain processes dominate in particular areas?
3. Which particular functions do these dominant processes have in communicating the speakers’ intentions?

### 1.1 Literature review

Language is employed by politicians to achieve their desired objectives. Charteris-Black (2005) points out that political leaders–since recorded history–have always employed linguistic forms to assure the public of the gains/advantages that would result from their leadership. This manipulation of language is not exclusively used by democratic or autocratic forms of government. Rather, it is a common characteristic and tactic of politicians in all the existing forms of government around the world. American presidential candidates, Hillary Clinton and Donald Trump, were no exception. Their third and last presidential debate before the 2016 presidentialelection is replete with evidence of such language manipulation from the comparative textual analysis through the transitivity framework of systemic functional grammar.

#### 1.1.1 Transitivity in traditional grammar

In traditional grammar words are divided into part of speech categories, of which one of the Category is of verbs. Verbs are usually divided into three categories which are

1) Transitive Verbs.
2) Intransitive Verbs.
3) Copular verbs.

In traditional terms, transitivity explains the phenomenon of either having the object after the verb or not. A verb is called transitive if it takes an object after it and it is called intransitive if it does not need an object after it. So borrow and enjoy are transitive verbs in “Ahmad borrowed some money from Shan” and “Sana enjoyed the party” since they need an object along with a subject. Whereas laughed and sit are the examples of intransitive verbs in “She laughed” and “He sat on sofa”. Speaking from the systemic functional linguistics' perspective, the whole clause is considered in the transitivity analysis rather than just the verbs found in it. The participants involved in carrying out the action determine the category of the processes.

### 1.2 Theoretical framework

M.A.K. Halliday developed the linguistic theory of discourse called systemic Functional Linguistics. The network of choices for the communication of meaning refers to ‘Systemic’ in systemic functional linguistics. Functional in the Hallidian model points towards the ways language operates in. According to Halliday (1967) language has three meta-functions: ideational, interpersonal and textual. The use of language determines context and vice versa as per Hallidian model of grammar.

Hallidyan Functional Grammar conceives language as a practical-cum-functional source for the communication of meanings. In a text, all the three meanings are articulated and communicated at the same time as per systemic functional grammar model by Halliday yet each meta-function retains its singularly individual meanings. This study seeks to explore experiential meaning in ideational metafunction. The ideational meta-function encompasses the experiential and logical meanings. The former uses transitivity framework for the interpretation of a text whereas the latter draws on clause complex to meaningfully explore the text. The interpersonal meta function is centered on system of mood and modality in grammar. Theme system is the focal point of the textual meta-function. Participants, processes and circumstances make up the structure of a clause in ideational meta-function and the present study has been designed within the canvass of ideational meta-function. Employing transitivity system, choices of processes in the text by each candidate are explored with a view to describe and comparatively analyze their world of experiences through determining the.process types and their relative frequency of occurrence.

Meanings are made according to SFL by the co-working of vocabulary and grammar. Lexico-grammar is the combined form of lexis and grammar. Expression of meanings in systemic functional grammar is articulated through three meta-functions: Ideational, Interpersonal and Textual. The ideational meta-function is related to the tangible as well as intangible articles and events in both the concrete and abstract worlds of experience. Two integral parts of this function are experiential and logical. The present study is to do with the former part of the meta-function which focuses on this world's knowledge and experience. The second meta-function deals with interactants’ relationships. The third meta-function is concerned with textual making up and coherence. All the three meta-functions contribute at the same time in the communicative events and are not worked out in isolation. Three corresponding parameters of context with the meta-functions are Field, Tenor and Mode.

Several salient specifications symbolize the field of the discourse. First of all, it is the lexis that helps identify the field of any textual construction. For example, David's lovely pet, his cat, a Spanish-born animal, her furred skin, active tail, her sense of smelling, her cute appearance, silent gait and faithful proximity are the tokens and samples of the lexis which help establish the semantic relation of the lexis to the topic ‘David's pet’. Similarly, the field of political discourse is easily identifiable as well.

SFL takes clause as its fundamental scale for analysis. Processes, participants and circumstances make up a clause in the Hallidian model of functional grammar as is shown in Table 1. Processes are such groups as are verbal ones and the focal point of the current study so far as the answers to the research questions are concerned. A clause cannot exist independently without a process whereas it can without a circumstance. The number of participants varies from clause to clause depending on the type of the process used in the clause. There may be one or two or three participants in a single clause.

**Table 1:** Sample clause in systemic functional grammar.

Participant	Process	Circumstance
She	Slept	at five O'clock.

Note. The basic unit in Systemic Functional Grammar is clause.

According to Halliday, six key process-types include material, mental, behavioral, verbal, relational and existential.

#### 1.2.1 Mental processes

Mental process types are found in clauses dealing with cognition, perception, understanding and affection. Impress, see, like, please and meditate are examples of mental process type verbs. The range of participants in this type of clauses is limited and well-defined. A sensor (a human like) and a phenomenon are usual participants as is shown in Table 2.

**Table 2:** Sample clauses containing mental processes.

Sensor	Process	Phenomenon
David	Saw	his teacher.
Christopher	Thought	that Hassan was going.
Jane	Liked	what her husband liked.

#### 1.2.2 Material processes

Clauses having material processes relate physiological actions and movements. Comb, catch, throw, walk and bite are the examples of material processes or verbs. Participants in the material process-type clauses may be actors, goals, recipients or beneficiaries as is shown in the following Table 3.

**Table 3:** Sample clauses containing material processes.

Actor	Process	Goal	Circumstance
Ayesha	Drives	her sister	home.
Goal	Process	Circumstance	Actor
Her sister	Is driven	home	by Ayesha.
Actor	Process	Recipient	Goal
Ayesha	Gave	her sister	a book.
Actor	Process	Beneficiary	Goal
Ayesha	Built	her sister	a house.

#### 1.2.3 Behavioral processes

Behavioral process types examples are: weep, breathe, stare, laugh and sneeze as is shown in Table 4. Material and mental type processes are semantically and characteristically combined for the formation of these process types. Such processes are the physiological actions resulting from psychological or mental functions. They are also called intermediate processes. Behaver is the sole participant which helps the clause perform its function.

**Table 4:** Sample clauses containing behavioral processes.

Behaver	Behavioral process	Circumstance
Mary	Laughed	at my folly.

#### 1.2.4 Verbal processes

Verbal process types serve to communicate ideas and feelings by saying something. The key participants of such clauses are sayer, addressee and verbiage as is shown in Table 5. There are various instances of talking, calling and saying many things to the fellow speakers in the everyday life and these constitute verbal type of processes.

**Table 5:** Sample clauses containing verbal processes.

Sayer	Process	Addressee	Verbiage
Ayesha	Told	Umar	to go
Addressee	Process	Verbiage	Sayer
Umar	was told	to go	by Haleema

#### 1.2.5 Relational processes

Relational processes are concerned with the process of being in the world of abstract relations (Thompson, 2004). Relational process clauses are of two types: identifying and attributive. They express qualities, possession and description. Existence and being of something at somewhere are communicated through relational process clauses. The ‘Be’ verb is most commonly used to express such type of processes. The participants in identifying relational process type clauses are token and value whereas in attributive clauses they are carrrier and attribute as is evident in Table 6.

**Table 6:** Sample clauses containing relational processes.

Token	Process	Value
Imran Khan	Is	Prime minister.
Carrier	Process	Attribute
Hassan	Is	smart.

**Table 7:** Sample clauses containing existential processes.

Process	Existent	Circumstance
There is	a lemon	in the basket.

#### 1.2.6 Existential processes

Existential process type clauses are usually represented through ‘there’. The existence is expressed through this empty category which takes the verb ‘be’. The sole participant in existential type clauses is ‘existent’ as can be seen in the following table.

Fairclough (2000) claims that politicians make use of language or choose particular linguistic forms to argue, reason, sustain their ideas, perpetuate their perspectives, prolong their tenure of power, denigrate people and peoples, advocate the public needs that are in their own interests. A functional analysis of the text of the sample debate in the present research cannot be made without exploring the reasons for the distribution and the occurrence of particular linguistic forms in six segments of the debate

Zhang (2017) argued in his study that political speakers expressed themselves in a very calculated manner making such choices of words as are extremely beneficial to their interests. He employed the transitivity model in Halliday's SFL so as to conduct an analysis of the very first television debate between Trump and Hillary. His focus was on quantitative analysis. He strived to explore the differences and similarities in the distribution of process types by the two candidates. He found as well the stimuli and functions of these distributions which convey their intentions.

The researcher obtained and compared the category-wise number of the six process types in the first television debate between the presidential candidates. The findings revealed that material processes, relational process and mental processes dominated the text. Trump used far more existential processes than Hillary Clinton. He concluded that material processes were attributed to strength and determination as well as to the actions of the new president and the government

The text analyzed in that study was one of the series of the debates which would be our focus in the present study. That study elaborated the prelude to the presidential candidates’ intentions whereas this research venture explained, by quantitatively and qualitatively analyzing the third and final American presidential debate, the summative outlooks and efforts made in the campaign for presidency.

Zhu Yujie & Li Fengjie (2018) tried to explore the deep social significance via transitivity analysis of the ideational function. The analysis showed that In Donald Trump's Inaugural Address the material process was the most frequently used process. The relational process and the mental process took the second and the third position respectively. In transferring power to the people, material process was mostly involved. While advocating for U.S. interests in international contacts, relational process topped the list of all the six processes. In recovering the audience's confidence and looking forward to a better future, American President Donald Trump applied different mental processes.

Based on transitivity system in Halliday's Systemic-Functional Grammar, Mengyan ZHAO1 and Yi ZHANG conducted transitivity analysis of American president Donald J. Trump's inaugural address in 2017. A mix method approach was used to explore the distribution and functions of six transitivity process in the inaugural address. They found that among the six processes, material processes (68.6%) highly dominated the speech. Relational processes (15.7%) ranked the second, followed by existential processes (6.4%), behavioral processes (5.0%) and mental processes (3.6%), while verbal processes (0.7%) seldom appear in the speech. The researchers also provided the different functions of each process. President Trump frequently used material processes to paint a bleak picture of America and to describe new actions the government will take.

He also used relational processes to lay out a new vision for America.

Mengyan (2017) set out to investigate the transitivity choices made by Donald Trump in his inaugural speech in 2017. The distribution and functions of the verbal groups were looked at in the study. A mixed method approach was taken for the analysis in this study. The results demonstrated that material processes made up about sixty nine percent of the processes used and thus dominated the text. The functions of all process types (material and mental) were substantially illustrated using the same method in the present study.

Junling (2010) argued in his study of two Barack Obama presidential speeches that politicians win public support through their manipulation of language. The researcher critically analyzed the texts using Halliday's systemic functional grammar. The results revealed that in both speeches, material processes dominated. Mental, relational, behavioral, verbal and existential process types respectively, were present in the text

Naz (2012) investigated the linguistic skills of Benazir Bhutto in her political speeches. Her manipulation of language was explored by the researcher in terms of her with transitivity choices. She concluded that word-play was a particular characteristic of the former prime minister of Pakistan.

Amma (2015) qualitatively analyzed the manipulation of language by Ghana's President, John Evans Attah Mills, in his maiden State-of-the-Nation speech. Content analysis of the text was carried out to investigate the communicative benefits and purposes of the linguistic choices made. 536 clauses were analyzed, and the findings revealed that about 60% of process choices were material. This suggests that Mills and his government were the main actors in the clauses. Mental processes were the least used, which means that Mills extended very few promises and assurances to the people, but rather, narrated and explained things as they were. Sofia (2011) investigated the linguistic manipulation in three important speeches by Barrack Obama delivered within the span of six years. The aim was to study the factors involved in convincing the public to support him. These factors embedded in his speeches were revealed through the linguistic choices Obama made to achieve his aims. The results of the textual analysis indicated that relational processes were used by Barrack Obama to paint a positive picture of him in the minds of the listeners. Material and mental processes were used to urge people physically to support him and emotionally engage with him in the 2008 American presidential general elections.

Mohammad (2017) conducted an analysis of Donald Trump's acceptance speech in the American presidential election 2016. The linguistic choices made by Trump were revealed through using Norman Fairclough’s critical discourse analysis model. Mohammad's aim was to determine the strategic use of language by Trump as presidential candidate during the election campaign. The results of this study revealed that Americanisms, immigration, terrorism, the rigged system, the economy, trade and foreign policy were central concerns for Trump

### 1.3 Research Methodology

COMSATS University has some approving committee members, part of COMSATS Ethical Committee that have approved that the study complies with all regulations and consent was taken from all the participants of study. The transcript of the third debate was selected for analysis as it is considered the best and the most effective illustration of the use of language by Hillary Clinton and Donald Trump of the three debates. Chris Wallace of Fox News hosted the debate and this debate was sponsored by the Commission on Presidential Debates. This commission had designed the format of this debate and Chris Wallace decided the topics and the questions from six areas which form the core of the American political landscape. These six areas are:

i The Supreme Court
ii Immigration
iii The economy
iv Fitness for the presidency
v Foreign hotspots
vi The national debt

Mixed methods were chosen for this study, considered the most suitable design given the goals of the study. Mixed methods are employed when more than one research design is necessary to study a problem. To measure the frequency of the six process types in each area of the debate in order to describe the candidates’ language use tendencies demands an interpretive research strategy.

The present study employed both quantitative and qualitative methods to achieve the research objectives of identifying and determining the category of process-types as well as their frequency in political discourse using a systemic functional Grammar framework. The factors which stimulated the American presidential candidates in their choice of verbs in six distinct segments of the text were investigated to identify variations in the frequency of process-types in the final presidential debate 2016.

In order to answer the research questions, six tables demonstrate the frequency of certain processes in each area of debate by each candidate. Thereafter, a cumulative table presents all the data obtained.

### 1.4 Data analysis

The reasons and functions underlying these choices and how they were enacted were unfolded through the qualitative analysis of the process types each speaker used and then comparing Clinton and Trump’s percentage use of each process type through the Systemic Functional Grammar transitivity framework.

#### 1.4.1 Supreme Court segment

Table 8 is followed by a discussion of each process type and its use by the speakers.

**Table 8:** Supreme Court segment.

Process Types	Hillary Clinton	Donald Trump	Clinton's percentage	Trump's percentage
Mental	58	28	67%	33%
Material	36	20	64%	36%
Behavioral	18	02	90%	10%
Verbal	14	15	48%	52%
Relational	61	60	51%	49%
Existential	07	00	100%	0%
Total	194	125	61%	39%

##### 1.4.1.1 Mental processes in the Supreme Court segment

Hillary Clinton used mental processes more frequently than Donald Trump, 67% compared to only 33%. This considerable difference is due to her serious cognitive and passionate concerns concerning the issue of the Supreme Court in contrast to Trump. Clauses such as “*I understand (mental) that Donald has been strongly supported (mental) by the NRA*”, “*That's how I see (mental) the court”,* “I would hope (mental) that the Senate would do its job and confirm the nominee that *President Obama has sent to them.”* and “*I think (mental) we need (mental) comprehensive background checks*” point to the fact that she felt more strongly, thought more profoundly, perceived more acutely and cared about the judgments made by the judges or their selection process. The dominant use of mental process types by Hillary Clinton had the additional function of assuring the audience that she would reform the justice system if elected.

##### 1.4.1.2 Material processes in the Supreme Court’s segment

Hillary Clinton employed 64% of the material process types in this section while Trump only used 36%. Material process types were used to represent tangible actions. “*Dozens of toddlers injure (material) themselves even kill (material) people with guns because, unfortunately, not everyone who has loaded (material) guns in their homes takes appropriate precautions.*” The use of these process types suggests that she intended to make concrete initiatives for the reformation of the Supreme Court. On the other hand, Donald Trump lagged behind her in this area of debate. He tried to make the claims in material terms to convince the voters. As for example “And those are the people that I will appoint. (material)” Overall, Donald Tump's argument contained fewer material process type statements in this area of the debate.

##### 1.4.1.3 Behavioral processes in the Supreme Court segment

Hillary Clinton used more behavioral process types than Donald Trump, 90% and 10% respectively. Clinton's *“And the court did not accept(behavioral) that reasonable regulation but they've accepted(behavioral) many others.”*, and “*We stand (behavioral) up against Citizens United, we stand (behavioral) for the rights of people in the workplace*.” could be as representative example. The underlying reason for Clinton's dominant use of behavioral process types was her previous constant and prolonged discussions on the Supreme Court issue in the second presidential debates, which enabled her to make an intermediate and conditioned response. Trump's limited use of behavioral process types suggests that his interest was more in revolutionary and drastic changes in the arms-bearing laws his interpretation of the constitution and his views on the appointment of judges to the Supreme Court.

##### 1.4.1.4 Verbal processes in the Supreme Court segment

Trump used slightly more verbal process types than Clinton, 52%–48%. He used these process types to compare his claims to those of his political rival, for instance, on abortion: “*If you go with what Hillary is saying (verbal), in the ninth month, you can take a baby and rip the baby out of the womb of the mother just prior tothe birth of the baby. Now, you can say (verbal) that is okay, and Hillary can say (verbal) that that is okay, but it's not okay with me*.” and “*But these were statements that* should never, ever have been made (verbal).”

Making claims is part and parcel of the election campaign. Trump made various claims using verbal process types. Trump's prime aim was getting his claims endorsed by calling upon a host of *reliable* sources. Referring to the second amendment and similar issues, he intended to turn voters against Clinton and simultaneously elicit favorable responses from the audience.

##### 1.4.1.5 Relational process types in the Supreme Court segment

Clinton used 51% relational process types whereas Trump used 49%. Here the difference is not striking. Clinton evaluated events and persons, telling people how things were and what she was going to do after being elected. “It really raises (relational) the central issue in this election. Namely, what kind of country are we going to be (relational)? What kind of opportunities will we provide (relational) for our citizens? What kind of rights will Americans have (relational)?” Hillary Clinton was found to be comparatively good at evaluating events and persons. She was painting such a picture, by means of using relational process types excessively, as stood her in good stead by telling people as to how things were and what she was going to do with them after being elected.

##### 1.4.1.6 Existential process type in the Supreme Court segment

Trump used no existential process types to refer to issues relating to the Supreme Court. Clinton used seven existential process types through her formal style and tone of communication. “There can be (existential) and must be (existential) reasonable regulation.” The existence of objects and their preliminary introduction is a feature of Clinton's speech.

#### 1.4.2 Immigration segment

Table 9 is followed by a discussion of each process type and its use by the speakers.

**Table 9:** Immigration segment.

Process Types	Hillary Clinton	Donald Trump	Clinton's percentage	Trump's percentage
Mental	37	47	44%	56%
Material	61	65	48%	52%
Behavioral	10	13	43%	57%
Verbal	23	19	55%	45%
Relational	71	76	48%	52%
Existential	02	01	67%	33%
Total	204	221	48%	52%

##### 1.4.2.1 Mental process types in the immigration segment

Clinton used mental processes less frequently when commenting on immigration than Donald Trump, 44% to his 56%. He expressed his views on immigration in unequivocal terms and more emphatically, as evidenced from “*We need (mental) strong borders*.” and “They know (mental) it better than anybody.” Trump used More mental process types because border security was a key issue for him, and he advocated building a wall to minimize the entry of foreigners and drugs. His ideas on border security appeared to be inflexible and he realized how sensitive the issue of defending the country was and had more workable plans in mind than Clinton. His higher percentage of mental process type use also reflected his before time perception of the consequences the country might face if what he sees as appropriate measures were not taken.

##### 1.4.2.2 Material process types in the immigration segment

Donald Trump used 52% material processes in this area of debate whereas Hillary Clinton used 48%. Examples of Trump's employment of material process types are: “*Children have been killed, (material) brutally killed (material), by people that came (material) into the country illegally.*” and “*They're coming in (material) illegally. Drugs are pouring in (material) through the border.”* Just as his use of mental process types, Trump's approach was more tangible and practical on immigration as evidenced by his extensive use of material process types.

##### 1.4.2.3 Behavioral process types in the immigration segment

Donald Trump extensively used behavioral processes in this area of debate to emphatically respond to American nationality holders' concerns by comprehensively combining mental, sensational and physical initiatives. Trump used 57% behavioral process types whereas Clinton used 43%. While talking about immigrants Trump told that U.S. Immigration and Customs Enforcement agency has endorsed him. “*First time they've endorsed (behavioral) a candidate.”* Trump was found more dominant on the issue of immigration for he never allowed the least interference from outside (from immigrants) as to surface from the preceding quoted clause. Trump used more behavioral process types' than Clinton, just as he did the same in the two preceding categories.

Clinton's seemingly more flexible and tolerant outlook was communicated through her use of fewer behavioral process types. In this portion, Clinton's marginal use of this type had the function of welcoming non-mainstream voters to support her, while Trump greater focus on behavioral process types appealed to the mainstream American prejudice against outsiders at this critical juncture in the presidential election procedure

##### 1.4.2.4 Verbal process types in the immigration segment

56% verbal process types were used by Hillary Clinton, 45% by Donald Trump drawing She used more verbal process types in response to Trump criticizing her for being the outsiders' advocate. She criticized his stance and the bias of others, saying, “*He basically said (verbal) what a lot of employers do (verbal)*.”, “*But you are very clearly* quoting (verbal) from WikiLeaks”.

Through using verbal process types, Clinton defined her relationship with Putin, the Russian leader, in the political arena. She used verbal process types to assure listeners she would resume and retain her policy on immigration and would facilitate immigrants’ lives if elected

##### 1.4.2.5 Relational process types in the immigration segment

Trump used 52% of the relational process types in this area whereas Hillary Clinton used 48%. Trump's slightly larger use was due to his descriptive techniques used to refer the current policy of immigration. *“You have (relational) thousands of* mothers and fathers and relatives all over the country.” “The biggest complaint they have (relational), it's (relational) with all the problems going (relational) on in the world, many of the problems caused (relational) by Hillary Clinton and Barack Obama”

Relational clauses are of two types: identifying and attributive. The attributes of the current foreign policy were vehemently described by Trump through the recurrent use of relational process type. The *outsiders'* identity was also determined by Trump through the process types of being and existing.

##### 1.4.2.6 Existential process types in the immigration segment

Hillary Clinton used 67% existential process types whereas Trump used 33% in this area. Existential processes are processes of existence. The presence of many immigrants in the United State was justified in existential terms, for example, in the following claim. *“There are (existential) some limited places”.* Another reason behind Clinton's greater use of existential processes on the topic of immigration was her acceptance of immigrants. Trump, by contrast, discouraged the acceptance of immigrants. The existential reference above had an empty category ‘there’, such use was mainly by Clinton to communicate the immigrants' unrecognized existence.

#### 1.4.3 The economy segment

Table 10 is followed by a discussion of each process type and its use by the speakers.

**Table 10:** The economy segment.

Process Types	Hillary Clinton	Donald Trump	Clinton's percentage	Trump's percentage
Mental	35	29	55%	45%
Material	52	76	41%	59%
Behavioral	03	08	27%	73%
Verbal	12	28	30%	70%
Relational	98	72	58%	42%
Existential	01	00	100%	0%
Total	201	213	48%	52%

##### 1.4.3.1 Mental processes in the economy segment

Clinton used 55% of the mental process types in the Economy segment, while Trump used45% mental process while talking about the Economy. Clinton's greater use reflected her approach to developing the economy of the country and analysis of Trump's. plans “*I want (mental) more technical education …...”* “*Donald's plan has been analyzed (mental) to conclude (mental) it might lose (mental) 3.5 million jobs*.”Her knowledge of the facts and figures and economic insights the have resulted in her using more mental process types.

##### 1.4.3.2 Material processes in the economy segment

59% material process types were used by Trump to answer questions related to economy and 41% by Clinton. The more frequent use by Trump reflects his concrete schemes to boost the declining economic condition in the USA. Unlike Clinton, he looked for ways to reduce labor costs with greater returns on investment. His employment plans proved to be more workable which could be gauged from the following clauses uttered by him. “*We protect (material) Saudi Arabia. Why aren't they paying (material)?” “They're going to start (material) hiring people*.”

Trump emphasized the need for a physically strong and stable economy, which received a favorable response from the public, his main objective as a political leader.

##### 1.4.3.3 Behavioral process types in the Economy segment

Trump made greater use of behavioral process types than Clinton, 73% compared to 27%. This dominance accounted for his two-dimensional approach to economy of the country. He believed in the comprehensive and logical vision along with physical movements to achieve economic prosperity. His argument contained the following: “*Boy, did they suffer (behavioral)?” “You look (behavioral) at her real record.”* He saw bilateral relationships as the best way to develop the American economy.

##### 1.4.3.4 Verbal process types in the Economy segment

Trump used 70% verbal process types in this area whereas Clinton used 30% of the total. Trump's use of verbal process may be due to his inexperienced approach and slogans, having not held office so with nothing but words to rely on. *“I questioned* (verbal) it, and I questioned (verbal) NATO, why aren't they NATO questioned *(verbal)?”* Budgetary schemes were described through mainly verbal process types, which were also used to draw public attention to the questionable deals of the previous government and winning voters’ appreciation

##### 1.4.3.5 Relational process types in the Economy segment

Hillary Clinton used 58% of the overall relational process types in remarks devoted to the economy, while her political rival used forty-two percent of them. *“The* middle class thrives (relational), America thrives (relational). So my plan is (relational) based on growing the economy.” “His whole plan is (relational) to cut (relational) taxes.” “When my husband was (relational) president, we went (relational) from a $300 billion deficit to a $200 billion surplus, and we were (relational) actually on the path to *eliminating the national debt.”* Clinton's comments were based on identifying the key factors for economic growth and using facts and figures as an evidence of her husband's achievement as president.

##### 1.4.3.6 Existential process types in the Economy segment

Only one existential process type clause was uttered by Clinton. *“There's* (existential) only one of us on this stage who has actually shipped jobs to Mexico *because that's Donald.”* Her use of this existential process type was to emphasize Trump's *unpatriotic* behavior. It resulted from her concept that Donald had emptied their country of jobs by sending them to Mexico. She used an empty category ‘there’ to express it.

#### 1.4.4 Fitness to be president segment

Table 11 is followed by a discussion of each process type and its use by the speakers.

**Table 11:** Fitness to be president segment.

Process Types	Hillary Clinton	Donald Trump	Clinton's percentage	Trump's percentage
Mental	35	55	39%	61%
Material	56	60	48%	52%
Behavioral	06	11	35%	65%
Verbal	31	29	52%	48%
Relational	81	78	51%	49%
Existential	05	02	71%	29%
Total	214	235	48%	52%

##### 1.4.4.1 Mental process types in the fitness to be president segment

Mental process types were used more frequently by Trump than Clinton in response to the question of their fitness for the presidency, 61% compared to 39%s. Trump's utterances in this segment reflect his mental, intellectual, emotional and perceptions of his fitness to be president “*And you know (mental) it and they know* (mental) it and everybody knows (mental) it.” “But unfortunately for them, I think (mental) the voters are seeing (mental) through it. I think (mental) they're going to see (mental) through it, we'll find (mental) out on November 8th, but I think (mental) they're going to see (mental) through it.”

His use of this process type reflects his seeming greater confidence – real or portrayed – and confidence than Clinton expressed, in being elected the next president of the United States of America. He also aimed to convince the public of his suitability by using their own sense reality rather than believing the statistical data provided by the current government

##### 1.4.4.2 Material process types in the fitness to be president segment

Trump once again used slightly more material process types (52%) than his political rival (48%). Trump questioned the previous government's policies to refute the claims of his rivals. *“Why don't you give (material) back the money that you've* taken (material) from certain countries that treat (material) certain groups of people so horribly?” “I don't buy (material) boats. I don't buy (material) planes.”

##### 1.4.4.3 Behavioral processes in the fitness to be president segment

Trump made 65% of the behavioral process types in this segment, in his responses. Trump's behavioral process type utterances suggest his physical actions had been and would be pregnant with rationally-based. *“I will look (behavioral) at it (election results)* at the time. I'm not looking (behavioral) at anything now, I'll look (behavioral) at it at *the time.” "The New York Times actually wrote (behavioral) an article about it”.* These behavioral process types illustrate his current and longer-term stance on the fairness of elections. Such behavioral process types are characteristic of Halliday's model.

##### 1.4.4.4 Verbal processes in the fitness to be president segment

Hillary Clinton used 52% percent of the verbal process types in this segment while Trump used 48%. While talking about Trump, she stated: *“He called (verbal) her* disgusting, as he has called (verbal) a number of women during this campaign.” “He *never apologizes (verbal).”* Clinton criticized Trump’s unscrupulous and stubborn attitude to stress his unfitness for the presidency.

##### 1.4.4.5 Relational processes in the fitness to be president segment

Clinton expressed through 51% of the relational process types while Trump used 49% relational processes in the whole discussion. Relational processes are used to identify a person, quality, state or their relationship, or to attribute certain qualities to certain people or objects. Clinton stated: *“It****'****s (relational) really up to all of us to* demonstrate (relational) who we are (relational) and who our country is (relational).” “It really does come (relational) down to what kind of country we are going to have (relational).”

Clinton's relational process types in this segment referred to self-identification and the prospect of realizing the implications, as part of her strategy to win credit for being open and rational. Trump used relational processes while defending himself from the allegations put on him *“Well, first of all, those stories have been largely debunked* (relational)” OR “we had (relational) such violence”

##### 1.4.4.6 Existential process types in the fitness to be president segment

71% of the existential process types in this segment were in Clinton's utterances, contained within her argumentative tone, as illustrated by the following: “They concluded there was (existential) no case*.” “There was (existential) even a time* when he didn't get an Emmy for his TV program.” “Of course, there's (existential) no *way.”* She was distinguishing between fake cases and real incidents and the real ones, to influence voters’ perceptions to persuade them to vote her in the upcoming elections, by her use of evidential affirmation and negation.

#### 1.4.5 Foreign hotspots segment

Table 12 is followed by a discussion of each process type and its use by the speakers.

**Table 12:** Foreign hotspots segment.

Process Types	Hillary Clinton	Donald Trump	Clinton's percentage	Trump's percentage
Mental	35	42	45%	55%
Material	45	48	48%	52%
Behavioral	09	10	47%	53%
Verbal	12	12	50%	50%
Relational	72	96	43%	57%
Existential	02	00	100%	0%

##### 1.4.5.1 Mental processes in the foreign hotspot segment

Trump used 55% of the mental process types in the foreign hotspot’ segment Trump's outlook, calculations and wishes were communicated using mental process types. *“I agree (mental) with both.” “What do you need (mental), a signed document?” “I mean (mental), cash, bundles of cash as big as this stage.”* Trump aimed to communicate to the audience his sincerity, vigilance and that other stakeholders who endorsed his proposals as well.

##### 1.4.5.2 Material process types in the foreign hotspots segment

Trump used 52% material process types to share with public the physical repercussions of policy initiatives in Iraq and Iran in the following utterances:. “We *gave (material) them $1.7 billion in cash.” “And they are being slaughtered (material) because of bad decisions.” “Iran is taking (material) over Iraq.”* His aim was to highlight actions causing losses to the national economy while allowing other countries to reap benefits.

##### 1.4.5.3 Behavioral process types in the foreign hotspots segment

Trump used 53% of the behavioral process types in this segment to suggest he had a shrewd, fact-based understanding of foreign hotspot issues. This intermediate concept is characteristic of behavioral process types, and reflected in the following clauses. *“I've been reading (behavioral) about going after Mosul.” “Iran should write (behavioral) us yet another letter.”* Trump juxtaposed his view of the ideal and beneficial behavior with harmful and unacceptable behavior in foreign policy matters.

##### 1.4.5.4 Verbal process types in the foreign hotspots segment

Interestingly, each text producers used half of the total verbal process types in his segment, Trump to divulge secrets and Clinton to clarify any confusion resulting from Trump's allegations, in the following: *“Let me tell (verbal) you.” (Trump), and* Clinton's response: “I said (verbal) that years ago. He has consistently denied (verbal).”

The choices of verbal processes by both speakers function as a position-clarifying tool and the link between past remarks and the present stances.

##### 1.4.5.5 Relational process types in the foreign hotspots segment

Trump used 57% of the relational process types to show the relationships, identities and attributes of the elements involved to win the public support by identifying the agents who caused disastrous incidents with nightmarish effects. For example *“All she had (relational) to do was(relational) stay(relational) there, now* we're going in to get it. But you know who the big winner in Mosul is going to be(relational) after we eventually get it”

##### 1.4.5.6 Existential process types in the foreign hotspots segment

As in other areas of the debate, existential processes were used mainly by Clinton, and 100% in this segment in two existential utterances: *“There is (existential)* an effort led by the Iraqi Army.” “There are (existential) several thousand fighters in *Mosul.”* She used existential process types to inform voters know she was aware of fighting in hotspots and of the countries whose interests were in encouraging this violence.

#### 1.4.6 National debt segment

Table 13 is followed by a discussion of each process type and its use by the speakers.

**Table 13:** National debt segment.

Process Types	Hillary Clinton	Donald Trump	Clinton's percentage	Trump's percentage
Mental	44	33	57%	43%
Material	20	44	31%	69%
Behavioral	04	01	80%	20%
Verbal	13	05	72%	28%
Relational	64	60	52%	48%
Existential	01	00	100%	0%
Total	146	143	51%	49%

##### 1.4.6.1 Mental processes in the national debt segment

Clinton used fifty-seven percent of the mental process types in this segment, as follows: *“I wonder (mental) when he thought (mental) America was great.” “I think* (mental) it's important to recognize (mental) that he has been criticizing (mental) our *government for decades.” “In fact, I think (mental) just the opposite.”* This suggests a claim of being comparatively aware of the sensitivity and pertinence of the issue of the national debt.

Political leaders play with the emotions of the public on critical issues and elicit their support by using mental process types such as ‘wonder’, ‘think’ and ‘feel’. Clinton strived to demonstrate her keen perception and views on the key issue of the national debt.

##### 1.4.6.2 Material processes in the national debt segment

69% of the material process types were in Trump's utterances, as follows: “*We're taking (material) back jobs.” “We don't make (material) our product anymore.”* “People, Chris, will again go (material) back to work, and they'll make (material) a lot *of money.”* Trump used material process types to suggest the national debt could be reduced by making the most of man-power, so his comments used process types related to physical effort and tangible actions. Trump had proved himself to be a successful businessman, therefore knew how to make idle people useful and contribute toe boosting the economy.

His use of material process types communicated the pragmatic and practical strategies he would put in place after being elected.

##### 1.4.6.3 Behavioral process types in the national debt segment

Clinton used eighty percent of the behavioral process types identified in this segment. The following are a few examples from the text. *“Well I would like to say to* everyone watching (behavioral) tonight.” “But if you look (behavioral) at the debt.” These comments illustrate her concerned attitude to the national debt and tangible aims to resolve the situation, hoping voters would to physically endorse her strategies (by voting for her) after considering them comprehensively.

##### 1.4.6.4 Verbal process types in the national debt segment

Clinton used 72% of the verbal process types identified in this area. For instance, *“A lot of the other issues that people talk (verbal) to me about all the time.”* “We are going to ask (verbal) the wealthy.” “I'll say (verbal) something about the *Affordable Care Act.”* Her dominant number of verbal processes while discussing the national debt accounted for her attitude of ‘give and take’. Furthermore, her convincing nature also became evident through it. Trump, on the other hand seemed less interested in dialogue and believed in flatly imposing policies, as suggested by his limited use of verbal processes in the national debt segment.

Clinton's use was in sharing her past achievements and making claims to grow the national economy in future.

##### 1.4.6.5 Relational process types in the national debt segment

As in the previous section, Clinton used 52% of the relational process types identified in this section*:* ***“****That's (relational) what my mission will be (relational) in the* presidency.**”** She identifies her optimistic plans to the voters:. “You have (relational) good jobs with rising incomes, that your kids have (relational) a good education from *preschool through college.”* These attributive relational process types were mainly used to help the voters visualize a prosperous future. *“That's (relational) part of my commitment to raise (relational) taxes on the wealthy.”* increasing taxation on the wealthy after becoming president.

Attributive relational process types were employed in order to attribute positive features to her own self and negative ones to her political rival.

##### 1.4.6.6 Existential process types in the national debt segment

Only, one existential clause was found in this section of the debate, uttered by Clinton: *“And there is (existential) no evidence whatsoever”.* Though this process type was used with an empty category ‘there’, physical and potential absence of evidential precedents was effectively communicated to the listeners. Since Hillary Clinton had been in the echelons of power, she corroborated her arguments by means of past developments. Donald Trump was unable to use such historical facts from political experience.

### 1.5 Cumulative analysis

The answers to three research questions were investigated. Clinton used more process types than Trump in the Supreme Court and national Debt's segments whereas Trump used more process types in the Immigration, Economy, Fitness to be President and Foreign Hotspot segments, but the overall difference was 51%–49%. However, Clinton used existential process types in all the six areas of the debate Table 14

**Table 14:** Cumulative analysis.

Six areas of debate	Hillary Clinton	Donald Trump	Total processes	Hillary Clinton’s percentage	Donald Trump’s Percentage
Supreme court	194	125	319	61%	39%
Immigration	204	221	425	48%	52%
Economy	201	213	414	48%	52%
Fit to be president	214	235	449	48%	52%
Foreign hotspots	175	208	383	46%	54%
National debt	146	143	289	51%	49%
Grand total	1134	1145	2279	49%	51%

### 1.6 Findings

The results reveal that, in the Supreme Court's segment, Hillary Clinton used all the process types more than Trump, except for verbal process types, where the margin of difference between speakers is narrow. She is the user of the only existential process type on the topic of the Supreme Court.

The frequent use of verbal process types by Trump comes from his tendency to make claims. On the other hand, Clinton communicated her intellectual, emotional, experiential, behavioral, attributive and evidential claims through her transitivity choices and aim to mold and win public opinion on the Supreme Court issue.

The findings in the immigration segment were that Trump used a more of the process types than Clinton except for verbal and existential process types. The difference is not great in the comparative analysis of transitivity choice frequency in this area of the debate. Existential processes are used more by Clinton in the immigration segment, as in the preceding segment. Trump's emphatic and frequent use of the four types of processes mainly shows his unequivocal attitude towards outsiders, while Clinton has a soft spot for non-Americans reflected in her pleading tone, justifying their existence, embodied in her verbal and existential process types. Trump expressed his intention of tightening border security and minimizing foreign interference through the use of mental, material, behavioral and relational process types to inform the public of the iron fist he intended to use to deal with outsiders. This function of stimulating the public to rethink their views of the Immigration issue was also performed by Trump's transitivity choices.

In the Economy segment, Clinton used more of the identified mental, relational and existential process types than Trump, who made the main use of the material, behavioral and verbal process types. The arguments underlying relatively dominant use of three process types by each speaker are interesting. Clinton's use of existential process types is substantially greater, while the material, behavioral and verbal process types used by Trump reveal his intention to make tangible and practical initiatives to boost the economy, according to his claims. On the other hand, Clinton shared her prospective financial plans, their attributes and success in the past, present and hoped for, in future through her transitivity choices of mental, relational and existential process types

Fitness to be president of America was the main topic of the rest of the discussion in the debate. The transitivity choices suggest that Trump had a clearer vision of running the country, the pragmatic policies to implement and reasoned actions to take than his opponent, from his frequent use of mental, material and behavioral process types in this segment had a confidence winning aim for him as the prospective president of America. Clinton used verbal, relational and existential process types more frequently than Trump. Her intention was to convince the voters through her transitivity choices.

The findings reveal that in Foreign Hotspots segment, Trump used more mental, material, behavioral and relational process types than Clinton. Verbal process types were used by both candidates to similar degree. Relational process types were used by Clinton to identify positive achievements in hotspot areas by the previous government. Trump relational process type use came from his disagreement with Clinton's party's policies, external insurgencies and their causes. His aim was to attracting voters yet to decide whom to vote for to his vision through his dominant use of four process types.

Except for material process types, Clinton made the main use of mental, behavioral, verbal, relational and existential process types from result from her political experience as First Lady and her communication techniques. Trump's frequent use of material processes comes from his tendency to offer practical solutions to reduce the national debt.

### 1.7 Conclusion

The overall use of process types in the text, in response to the first research question, reveals that overall, Trump, used fifty-one percent of the process types identified, slightly more than Clinton. However, in the areas of the Supreme Court and National Debt, it was Clinton that used more process types, more frequently, answering question 2, while Trump used more process types in the segments on Immigration, the Economy, Fitness to be President and Foreign Hotspots, suggesting his stronger views on these issues. Considering their persuasive intentions embedded in their choice of process types and their functions, the findings of the present study suggest that Trump expressed himself in this debate more meaningfully to the electorate than Clinton, thus answering question no. 3 of this research.

The findings show that all six process types were used by the presidential candidates in their debate to varying degrees, while discussing the six topics of the debate. This difference in their level of use reflects their corresponding thinking and feeling patterns, political and socio-economic experience, stimuli, communication. skills, identities and standpoints. It could also be seen that the main aim, illustrated by the frequent user of certain process types, was winning the favor of the American voters in the 2016 presidential elections 2016.

## Declarations

### Author contribution statement

Aasia Nusrat and Shahnila Tariq: Analyzed and interpreted the data.

Farah Kashif and Farzana Ashraf: Conceived and designed the experiments; Analyzed and interpreted the data.

Abdul Raees and Rabia Farooqi: Contributed reagents, materials, analysis tools or data; Wrote the paper.

### Funding statement

This research did not receive any specific grant from funding agencies in the public, commercial, or not-for-profit sectors.

### Data availability statement

Data included in article/supplementary material/referenced in article.

### Declaration of interest’s statement

The authors declare no conflict of interest.

### Additional information

No additional information is available for this paper.

## Appendix A Supplementary data

new doc 2022-08-05 13.20.30-20220805135754 new doc 2022-08-05 13.20.30-20220805135754
