# Supplementary material for: A transitivity analysis of Hillary Clinton and Donald Trump’s third presidential debate
Source: Heliyon. 2022 Sep 9;8(9):e10518. doi: 10.1016/j.heliyon.2022.e10518 (PMC9479016; doi:10.1016/j.heliyon.2022.e10518)
Supplement: new doc 2022-08-05 13.20.30-20220805135754 [file mmc1.pdf]

It is to inform you that all the following authors of the article "A TRANSITIVITY ANALYSIS OF HILLARY CLINTON AND DONALD TRUMP'S THIRD PRESIDENTIAL DEBATE" agreed upon the sequence of author list mentioned in the cover letter and show their willingness to be the part of this study.

Dr. Farah Kashif
Assistant Professor, Kinnaird College for Women University, Lahore, Pakistan.
nihaasd@hotmail.com

Dr. Rabia Farooqi
Associate Professor Department of Psychology University of Central Punjab,
Lahore, Pakistan.
rabia.farooqi@ucp.edu.pk

Dr. Shahnila Tariq
Associate Professor Department of Applied Psychology UMT, Lahore Pakistan
shahnila.tariq@umt.edu.pk

Dr. Aasia Nusrat (Corresponding author)
Assistant Professor, COMSATS University, Islamabad Lahore Campus, Pakistan.
Email: aasianusrat@cuilahore.edu.pk
ORCID ID: 0000-0002-0653-4148

Dr. Farzana Ashraf
Assistant Professor, COMSATS University, Islamabad Lahore Campus, Pakistan.
farzana.ashraf@cuilahore.edu.pk

Abdullah Raees
University of Lahore, Lahore, Pakistan.
Email: abdullah.raees786@gmail.com

Scanned with CamScanner
